# Monogenic diabetes syndromes: Locus‐specific databases for Alström, Wolfram, and Thiamine‐responsive megaloblastic anemia

**DOI:** 10.1002/humu.23233

**Published:** 2017-06-01

**Authors:** Dewi Astuti, Ataf Sabir, Piers Fulton, Malgorzata Zatyka, Denise Williams, Carol Hardy, Gabriella Milan, Francesca Favaretto, Patrick Yu‐Wai‐Man, Julia Rohayem, Miguel López de Heredia, Tamara Hershey, Lisbeth Tranebjaerg, Jian‐Hua Chen, Annabel Chaussenot, Virginia Nunes, Bess Marshall, Susan McAfferty, Vallo Tillmann, Pietro Maffei, Veronique Paquis‐Flucklinger, Tarekign Geberhiwot, Wojciech Mlynarski, Kay Parkinson, Virginie Picard, Gema Esteban Bueno, Renuka Dias, Amy Arnold, Caitlin Richens, Richard Paisey, Fumihiko Urano, Robert Semple, Richard Sinnott, Timothy G. Barrett

**Affiliations:** ^1^ Institute of Cancer and Genomic Sciences College of Medical and Dental Sciences University of Birmingham Edgbaston Birmingham UK; ^2^ West Midlands Regional Genetics Service Birmingham Women's and Children's Hospital Edgbaston Birmingham UK; ^3^ Department of Medicine (DIMED) University of Padua Padua Italy; ^4^ Wellcome Trust Centre for Mitochondrial Research Institute of Genetic Medicine Newcastle University Newcastle upon Tyne UK; ^5^ Newcastle Eye Centre Royal Victoria Infirmary Newcastle upon Tyne UK; ^6^ NIHR Biomedical Research Centre at Moorfields Eye Hospital and UCL Institute of Ophthalmology London UK; ^7^ Centrum für Reproduktionsmedizin und Andrologie WHO Kollaborationszentrum EAA Ausbildungszentrum Universitätsklinikum Münster Münster Germany; ^8^ IDIBELL Hospital Duran i Reynals 3ª Planta Gran Via de L'Hospitalet 199 E‐08908‐ L'Hospitalet de Llobregat Barcelona Spain; ^9^ Centro de Investigación en Red de Enfermedades Raras (CIBERER) U‐730 Hospital Duran i Reynals 3ª Planta, Gran Via de L'Hospitalet, 199, E‐08908‐L'Hospitalet de Llobregat Barcelona Spain; ^10^ Departments of Psychiatry Neurology and Radiology Washington University School of Medicine St. Louis Missouri; ^11^ Department of Clinical Genetics University Hospital/The Kennedy Centre Glostrup Denmark; ^12^ Institute of Clinical Medicine The Panum Institute University of Copenhagen Copenhagen Denmark; ^13^ University of Cambridge Metabolic Research Laboratories Wellcome Trust‐MRC Institute of Metabolic Science Box 289 Addenbrooke's Hospital Cambridge UK; ^14^ School of Medicine, IRCAN, UMR CNRS 7284/INSERM U1081/UNS Nice Sophia‐Antipolis University Nice France; ^15^ Department of Pediatrics Washington University School of Medicine One Children's Place St. Louis Missouri; ^16^ IT Services University of Glasgow Glasgow UK; ^17^ Tartu University Children's Hospital Tartu Estonia; ^18^ Department of Metabolism University Hospitals Birmingham NHS Foundation Trust Queen Elizabeth Hospital Queen Elizabeth Medical Centre Birmingham UK; ^19^ Department of Paediatrics Medical University of Lodz Lodz Poland; ^20^ Alström Syndrome Europe Woodpecker Cottage Paignton S. Devon UK; ^21^ Association syndrome de Wolfram Residence Gauguin Grand‐Champ France; ^22^ Unidad de Géstion Clínica de Garrucha Área de Gestión Sanitaria Norte de Almería, Avd. Dra. Parra Almería Spain; ^23^ Birmingham Women's and Children's Hospital Birmingham UK; ^24^ Diabetes Research Unit Horizon Centre Torbay Hospital NHS Foundation Trust Devon UK; ^25^ Department of Medicine Division of Endocrinology, Metabolism, and Lipid Research Washington University School of Medicine St. Louis Missouri; ^26^ Department of information and computing systems The University of Melbourne Parkville Australia; ^27^ Cambridge Centre for Brain Repair Department of Clinical Neurosciences University of Cambridge Cambridge UK; ^28^ Genetics Section, Physiological Sciences Department, Health Sciences and Medicine Faculty University of Barcelona

**Keywords:** Alström syndrome, genotype–phenotype analysis, locus‐specific database, Monogenic diabetes, Thiamine‐responsive megaloblastic anemia syndrome, Wolfram syndrome

## Abstract

We developed a variant database for diabetes syndrome genes, using the Leiden Open Variation Database platform, containing observed phenotypes matched to the genetic variations. We populated it with 628 published disease‐associated variants (December 2016) for: *WFS1* (*n* = 309)*, CISD2* (*n* = 3)*, ALMS1* (*n* = 268), and *SLC19A2* (*n* = 48) for Wolfram type 1, Wolfram type 2, Alström, and Thiamine‐responsive megaloblastic anemia syndromes, respectively; and included 23 previously unpublished novel germline variants in *WFS1* and 17 variants in *ALMS1*. We then investigated genotype–phenotype relations for the *WFS1* gene. The presence of biallelic loss‐of‐function variants predicted Wolfram syndrome defined by insulin‐dependent diabetes and optic atrophy, with a sensitivity of 79% (95% CI 75%–83%) and specificity of 92% (83%–97%). The presence of minor loss‐of‐function variants in *WFS1* predicted isolated diabetes, isolated deafness, or isolated congenital cataracts without development of the full syndrome (sensitivity 100% [93%–100%]; specificity 78% [73%–82%]). The ability to provide a prognostic prediction based on genotype will lead to improvements in patient care and counseling. The development of the database as a repository for monogenic diabetes gene variants will allow prognostic predictions for other diabetes syndromes as next‐generation sequencing expands the repertoire of genotypes and phenotypes. The database is publicly available online at https://lovd.euro-wabb.org.

## 1 INTRODUCTION

Monogenic diabetes syndromes are characterized by glucose intolerance together with extrapancreatic features, and result from one or more defects in a single gene. There are about 40 different genetic subtypes identified so far, with an estimated prevalence of 2%–5% of all patients with diabetes (Schwitzgebel, 2014). The wide phenotypic and genetic heterogeneity poses significant problems for our understanding of disease mechanisms, and for providing prognostic information. This is compounded by the identification of diabetes syndrome gene variants of uncertain significance, in isolated diabetes through the widespread application of next‐generation sequencing (Alkorta‐Aranburu et al., 2014; Ellard et al., 2013; Philippe et al., 2015). There are no up‐to‐date variant databases for most monogenic diabetes syndrome genes; those that do exist contain limited historical variants on publicly available Websites (HGVS: http://www.hgvs.org/dblist/dblist.html, GEN2PHEN: http://www.gen2phen.org/data/lsdbs, LOVD: http://grenada.lumc.nl/LSDB_list/lsdbs, WAVe: http://bioinformatics.ua.pt/WAVe, ClinVar: http://www.ncbi.nlm.nih.gov/clinvar).

Wolfram (type 1, MIM# 222300; type 2, MIM# 604928), Alström (MIM# 203800), and Thiamine‐responsive megaloblastic anemia (MIM# 249270) syndromes are rare, monogenic syndromes where diabetes is a common feature. They are chronically debilitating, highly complex, and in common with other rare diseases, often subject to misdiagnosis, delayed diagnosis, and nondiagnosis. The syndromes exhibit clinical overlap: all can cause profound visual and hearing impairment, and diabetes mellitus (DM) or impaired glucose tolerance. With 0.57, 0.14, and 0.1 cases per 100,000 (Prevalence of rare diseases: Bibliographic data, 2013), all three syndromes also fall within the EU rare disease definition of “a prevalence of not more than 5 affected persons per 10,000 population” (Regulation (EC) No 141/2000 of the European Parliament, 2000).

Recommendations issued by the European Council in 2009 highlight the need for coordination and cooperation, and networking of resources throughout Europe (The Council of the European Union, 2009). A number of projects including Orphanet, EUROPLAN, and EURORDIS have made progress in this field. The EURO‐WABB project is an EU initiative to widen access to genetic testing, clinical information, and research for the overlapping rare diabetes syndromes Wolfram, Alström, Bardet Biedl syndrome, and others, in Europe (www.euro-wabb.org). As part of this project, we have created a new locus‐specific database to provide catalogs of gene variations involved in monogenic diabetes syndromes. By building on the existing generic frameworks and platforms for rare diseases, this gene variant database operates at a disease‐specific level to support efficient diagnosis and research for these syndromic diabetes diseases.

## 2 THE GENES

This report focuses on four genes in the EURO‐WABB locus‐specific database, namely *ALMS1*, *WFS1*, *CISD2*, and *SLC19A2*.

Pathogenic variants in the gene *ALMS1* (MIM# 606844) on chromosomes 2p13.1 have been identified in patients with Alström syndrome (AS), an autosomal‐recessive disease characterized by retinal dystrophy, childhood obesity, type 2 DM, and sensorineural hearing loss (Collin et al., 2002; Hearn et al., 2002; Marshall et al., 2005; Marshall et al., 2007; Marshall et al., 2015). Other features include dilated cardiomyopathy (70% of patients), hepatic diseases, and urological abnormalities (Alstrom, Hallgren, Nilsson, & Asander, 1959; Marshall et al., 2005; Marshall et al., 2007). *ALMS1* consists of 23 exons encompassing over 224 kb of genomic DNA encoding a centrosomal protein of 4,169 amino acids, which contain a large tandem‐repeat domain consisting of 47 amino acids and has been implicated in the assembly and maintenance of primary cilia (Hearn et al., 2005; Knorz et al., 2010; Li et al., 2007) and in fibrosis (Zulato et al., 2011).

Pathogenic variants in *WFS1* (MIM# 606201) cause Wolfram syndrome (WS) type 1, a rare neurodegenerative disease characterized by DM and optic atrophy (OA). The gene is located on chromosome 4p16.1, and codes for an 890 amino acid protein (Wolframin) consisting of eight exons spanning 33.4 kb of genomic DNA (Inoue et al., 1998). Wolframin is an endoplasmic reticulum (ER) membrane protein (Takeda et al., 2001), thought to function as ER calcium channel or a regulator of ER calcium channel activity (Osman et al., 2003) and is involved in the unfolded protein response via interaction with and regulation of the ER stress sensor ATF6α (Fonseca et al., 2010). It is under regulation by ER stress sensors PERK, IRE 1‐alpha, and ATF6‐beta (Fonseca et al., 2005; Odisho, Zhang, & Volchuk, 2015).

WS type 1 is also known as DIDMOAD due to the clinical features associated with the disease (diabetes insipidus, diabetes mellitus, optic atrophy, and deafness). Although nonautoimmune insulin‐dependent DM is the most common manifestation of WS, the most frequent cause of morbidity and mortality associated with the disease are neurological disorders and urinary tract complications (Kinsley, Swift, Dumont, & Swift, 1995).

Pathogenic variants in the *CISD2* gene (MIM# 611507) have been identified in patients with WS type 2 (Amr et al., 2007; Mozzillo et al., 2014). WS type 2 differs from type 1 in respect that so far no diabetes insipidus (DI) and psychiatric disorder has been associated with the disease, and the novel presence of defective platelet aggregation leading to peptic ulcer bleeding. *CISD2* is located on chromosome 4q24, and codes for a 135 amino acid protein ERIS (ER intermembrane small protein), which consists of three exons spanning a 64.7‐kb genomic region. ERIS is a highly conserved zinc finger protein of the ER membrane involved in the regulation of cellular calcium homeostasis and mitochondrial biogenesis (Wang et al., 2014). Immunoprecipitation studies showed that ERIS protein coded by *CISD2* does not interact with Wolfram protein (Amr et al., 2007). Studies in mice show that *cisd2* deficiency in these animals causes mitochondrial death and dysfunction accompanied by autophagic death (Chen et al., 2009). To date, only 13 individuals with *CISD2* mutations have been reported in the literature (Amr et al., 2007; Mozzillo et al., 2014; Rondinelli, Novara, Calcaterra, Zuffardi, & Genovese, 2015).

Thiamine‐responsive megaloblastic anemia syndrome (TRMA syndrome) is a rare autosomal‐recessive condition characterized by nonautoimmune DM (nontype 1), sensorineural hearing loss, and megaloblastic anemia. The gene responsible, *SLC19A2* (MIM# 603941), is located on chromosome 1q24.2, consists of six exons with 497 amino acids spanning a 22.5‐kb genomic region. It codes for a high affinity thiamine transporter (Diaz, Banikazemi, Oishi, Desnick, & Gelb, 1999; Dutta et al., 1999; Labay et al., 1999). Although anemia can be corrected by thiamine treatment, the hearing loss is progressive and irreversible. In TRMA syndrome patients, DM and hearing loss can manifest from infancy to adolescence.

## 3 THE DATABASE

We followed the guidelines for establishing locus‐specific databases (Celli, Dalgleish, Vihinen, Taschner, & den Dunnen, 2012; Vihinen, den Dunnen, Dalgleish, & Cotton, 2012). We formed a consortium of scientists and clinicians working with these diseases through the EU‐funded EURO‐WABB European Registry project (Farmer et al., 2013). The database is based on the Leiden Open‐source Variation Database (LOVD) platform V2.0‐36 (Fokkema et al., 2011) and stores both published and submitted variants.

Variations in the databases are named according to the HGVS nomenclature (Den Dunnen et al., 2016; http://varnomen.hgvs.org) and include descriptions at DNA and protein levels. Variants are numbered and described with respect to the NCBI reference sequences NM_015120.4 (NP_055935.4), NM_006005.3 (NP_005996.2), NM_001008388.4 (NP_001094344.1), NM_006996.2 (NP_008927.1) for *ALMS1*, *WFS1*, *CISD2*, and *SLC19A2*, respectively, with +1 = A of ATG start codon. Previously published variants that do not conform are renamed accordingly with the original description included in the entry to facilitate cross‐reference. Mutalyzer (Wildeman, van Ophuizen, den Dunnen, & Taschner, 2008) is used to verify variant description.

We included the following minimum data item set: pathogenicity, DNA change, genomic position in the reference sequence and genome assembly (GRCh 38), predicted protein change, mutation type, variant remarks (other information available for the variant), technique used, link to published reference if applicable, and the following anonymized clinical data: ethnic origin, gender, consanguinity, and clinical features. As standard for the LOVD system, the database has links to other services such as PubMed, HGNC, Entrez Gene, OMIM, and GeneCards, in addition to sequence databases. The databases catalogs variants identified in patients reported to have been diagnosed with AS, WS type 1/type 2, and TRMA syndrome.

In predicting variant pathogenicity, we followed guidelines from the American College of Medical Genetics and Genomics (ACMG) and the Association of Molecular Pathology (AMP) (Richards et al., 2015) and considered other supporting information such as experimental evidence, presence in multiple families, segregation with disease phenotypes, as well as the prediction algorithm SIFT (Ng & Hanikoff, 2003), and PolyPhen‐2 (Sunyaev et al., 2001).

The database is implemented on a secure server held by University of Melbourne, Australia, with only curators being able to modify contents. All components of the server service are checked regularly to ensure the long‐term integrity of the server and the information stored in it (LSDB software, operating system, back‐end database, and Web server). Submitted variants are accepted and published after curation. The databases are updated regularly, are publicly accessible (https://lovd.euro-wabb.org), and have been included in the Human Genome Variation Society (HGVS) list of locus‐specific databases. The summary of the types of variants stored in the *ALMS1*, *WFS1*, *CISD2*, and *SLC19A2* databases are shown in Table 1.

**Table 1:** Summary of the types of variants in *ALMS1*, *WFS1*, *CISD2*, and *SLC19A2* database

Gene	ALMS1	WFS1	CISD2	SLC19A2
Chromosomal location	2p13.1	4p16.1	4q24	1q24.2
Disease	Alström syndrome	Wolfram syndrome type 1	Wolfram syndrome type 2	Thiamine‐responsive megaloblastic anemia (TRMA) syndrome
Number of unique variants	268	309	3	48
Substitutions	133	208	2	30
Deletions	90	64	1	13
Duplications	34	28	0	2
Insertions	7	5	0	1
Indels	3	4	0	2
Translocations	1	0	0	0

The *ALMS1* database contains 268 unique variants, identified in 334 patients, including 17 previously unreported variants. The majority (49.6%; 133/268) are single‐nucleotide substitutions, of which 104 (78%; 104/134) of the base substitutions lead to codon termination. Deletions make up 34% (90/268) of the total variants reported (83 frameshift, six nonsense, and one in‐frame), with six variants having deletions of more than 10 bases. The rest of the variants are duplications (13%; 34/268), insertions (2.6%; 7/268), insertion/deletions or indel (1%; 3/268), and translocations (1/268). The majority of variants are reported in exons 8 (51.5%; 137/268), 16 (17.3%; 46/268), and 10 (16%; 43/268). The most frequently reported variant is the c.10775delC (p.Thr3592Lysfs6*) located in exon 16 that is reported exclusively in 28 patients with English ancestry. Missense variants are uncommon. Among the 24 missense variants in the *ALMS1* database, 11 (45.8%) are predicted to be pathogenic, four (16.7%) are likely pathogenic, four (16.7%) are benign or likely benign, and five (21%) are variants of uncertain significance.

The geographic origin of reported AS patients includes Europe (UK, Italy, Portugal, Sweden, Spain, France, Belgium, Germany, the Netherland, Norway, Serbia, Macedonia, Romania, Bulgaria, Slovakia, Yugoslavia, Poland, Ireland), North Africa (Morocco), the Americas (US, Canada, Brazil, Argentina, the Caribbean), and Asia and Oceania (Turkey, Israel, Iran, Iraq, Saudi Arabia, Lebanon, Japan, Taiwan, China, India, Korea, Melanesia). The gender of 194 patients is known and consists of 109 males (56.2%) and 85 females (43.8%).

The *WFS1* database currently contain 309 unique variants identified in 531 patients, including 23 previously unreported variants. The frequency of *WFS1* variant types consists of 67.3% (208/309) substitutions, 20.7% (64/309) deletions, 9% (28/309) duplications, 1.6% (5/309) insertions, and 1.3% (4/309) insertion/deletions. More than 50% (156/309) of *WFS1* variants are missense variants, 19% are frameshifts (60/309), and 16.5% are nonsense changes (51/309). In‐frame deletions, duplications, insertions, and indels made up 11.6% (36/309) of the variants, and eight (2.6%) affect putative splice or regulatory sites. Of the missense variants in the database, 57% (89/156) are predicted to be pathogenic, 32% (50/156) are likely pathogenic, five (3%) are benign/likely benign, and 7.7% (12/156) are variants of uncertain significance. Twenty‐nine of the missense variants are known to have an autosomal‐dominant mode of inheritance, 28 are involved in sensorineural hearing loss, and one variant (c.1385A>G; p.Glu462Gly) is associated with autosomal‐dominant congenital nuclear cataracts. Private variants (reported once or present in single family or small population) account for 43% (133/309) of the total unique variants in the *WFS1* database. The majority of the variants reported (86%; 264/309) are located in exon 8. Most commonly reported variants are c.1362_1377del (p.Tyr454*) reported 35 times in 18 patients, c.1243_1245del (p.Val415del) reported 33 times in 22 patients, and c.1230_1233del (p.Val412Serfs*29) reported 30 times in 26 patients. In 233 (44%) of WS patients in the database, variants occur in the homozygous state.

The geographic origin of WS patients reported includes Europe (UK, Italy, Germany, France, Denmark, Spain, the Netherland, Finland, Hungary, Poland, Russia), Americas (USA, Canada Brazil), North Africa, Middle East, and Asia (Lebanon, Iran, Iraq, Turkey, Japan, China, India, Pakistan), and Australia.

To date, the *CISD2* database contains three unique variants identified in 13 individuals, namely, c.109G>C (p.Glu37Gln), c.(103+1_104‐1)_(318+1_319‐1)del, and c.103+1G>A. All result in exon deletion of *CISD2* leading to early termination of the ERIS protein. The first *CISD2* mutation (c.109G>C, p.Glu37Gln) was identified in 10 family members of three consanguineous Jordanian families (Amr et al., 2007) who presented with DM, sensorineural hearing loss, optic neuropathy, peptic ulcer, and defective platelet aggregation. The variant caused missense changes in a conserved amino acid as well as aberrant splicing resulting in the deletion of exon 2. Recently, two *CISD2* mutations have been identified in patients of Italian origin, the c.(103+1_104‐1)_(318+1_319‐1)del and the c.103+1G>A, causing deletions of exon 2 and exon 1, respectively (Mozzillo et al., 2014; Rondinelli et al., 2015). The exon 2 deletion of *CISD2* is predicted to abolish the transmembrane domain of the protein.

Currently, there are 48 unique variants identified in 52 patients in the *SLC19A2* database. Most are substitutions (62.5%; 30/48) leading to missense (69%; 20/29) and early termination (27.6%; 8/29) of the SLC19A2 protein, with the possibility of transcript degradation via RNA‐mediated decay. The rest of the variants are deletions (27%; 13/48), duplications (4.2%; 2/48), insertions, and indels (6.3%; 3/48) causing frameshift and/or early termination of the SLC19A2 protein. Among the 20 missense variants in the *SLC19A2* database, 16 (80%) are predicted to be pathogenic, two (10%) likely pathogenic, and two (10%) variants of uncertain significance. Most of the reported variants are in exon 2 with c.697C>T (p.Gln233*) being the most commonly reported variant in TRMA patients originating from Iran and Turkey. From the 82 patients reported with TRMA syndrome, 27 (33%) are of Middle Eastern origin, 24 (29%) have originated from the Mediterranean, and 14 (17%) are from South Asia.

### 3.1 AS and WS patient recruitment and variant identification

Children and adult patients with AS were recruited to the DAS study (defining the phenotype in AS) (UKCRN 9044, REC approval 10/H0203/33). Children with AS, and children and adults with WS, were recruited to the EURO‐WABB European Registry study (UK REC approval 11/WM/0127). Appropriate informed consent was obtained from adult patients and parents/guardians of children. Assent was also obtained where possible from children under 16 years. Clinical histories and medical records were obtained for all participants.

Genomic DNA were extracted from peripheral blood lymphocytes using standard protocols. Sequencing of *WFS1* and *ALMS1* exons were performed using ABI 3730 automated sequencer (Applied Biosystems, Foster City, CA) after PCR amplifications. Identified variants were checked against dbSNP, 1000 Genomes Project, or ExAC (exac.broadinstitute.org) for the more recent samples.

### 3.2 Novel variants in *ALMS1* and *WFS1*

We report 17 novel germline *ALMS1* variants detected in 17 UK AS patients from 16 families and one Slovakian patient (Table 2) and 23 novel *WFS1* variants in 59 UK WS patients from 48 families (Table 3) referred to the West Midlands Regional Genetic Service, Birmingham Women's Hospital and Department of Medicine, Padua University, Italy. All novel variants identified were submitted to the EURO‐WABB database (https://lovd.euro-wabb.org).

**Table 2:** Genetic and clinical finding in Alström syndrome patients

Patient	Location	Nucleotide change^a^	Protein change	Gender	Clinical findings
ALSUK1	Exon 8 Exon 8	**c.4025_4026delinsA** **c.6325G>T**	p.(Gly1342Glufs*18) p.(Glu2109*)	F	Severely impaired vision, hearing difficulty requiring hearing aid, heart defect (infancy), obesity (infancy), raised creatinine (95 μmol/L), bladder dysfunction, chest infection, kyphoscoliosis.
ALSUK2	Exon 8 Exon 8	**c.4053_4054del** **c.4321C>T**	p.(His1351Glnfs*5) p.(Gln1441*)	M	Impaired vision (infancy), obesity (infancy), global developmental delay.
ALSUK3.1	Exon 8 Exon 8	**c.4225G>A** **c.4225G>A** **c.5081del** **c.5081del**	**p.(Val1409Ile)** **p.(Val1409Ile)** p.(Pro1692Leufs*39) p.(Pro1692Leufs*39)	M	Impaired vision (infancy), normal hearing, obesity (infancy).
ALSUK3.2	Exon 8 Exon 8	**c.4225G>A** **c.4225G>A** **c.5081del** **c.5081del**	**p.(Val1409Ile)** **p.(Val4091Ile)** p.(Pro1692Leufs*39) p.(Pro1692Leufs*39)	M	Impaired vision (infancy), hearing difficulty requiring hearing aid, Fallots tetralogy, obesity.
ALSUK4	Exon 10 Exon 8	**c.9258dup** c.5145T>G	p.(Asp3087*) p.(Tyr1715*)	M	Cardiomyopathy.
ALSUK5	Exon 18 Exon 18	**c.11738dup** **c.11738dup**	p.(Ser3914Lysfs*6) p.(Ser3914Lysfs*6)	F	Impaired vision (severe), heart abnormality (infancy), obesity (infancy).
ALSUK6	Exon 19	c.11881dup	p.(Ser3961Phefs*12)	M	Photophobia and nystagmus (8 yr) but not otherwise vision impaired, cardiomyopathy, hyperlipidemia, chronic renal failure, bladder dysfunction.
ALSUK7	Exon 19	c.11881dup	p.(Ser3961Phefs*12)	M	Photophobia and nystagmus (8 yr) but not otherwise vision impaired, hearing difficulties requiring cochlear implants, cardiomyopathy (50 yr), left bundle branch block, hyperlipidemia (55 yr), chronic renal failure (55 yr), bladder dysfunction
ALSUK8	Exon 5 Exon 8	**c.1011_1012del** c.6590del	p.(Cys337*) p.(Lys2197Serfs*10)	M	Impaired vision (severe), hearing difficulty requiring hearing aid, heart defect (infancy), DM (18 yr), NALFD, raised creatinine (81μmol/L).
ALSUK9	Exon 5 Exon 16	**c.800G>A** c.11107C>T	p.(Trp267*) p.(Arg3703*)	F	Impaired vision (infancy), normal hearing, cardiomyopathy (infancy), heart transplant then hemiparesis.
ALSUK10	Exon 8 Exon 16	**c.2224dup** c.10975C>T	p.(Thr742Asnfs*2) p.(Arg3703*)	F	Registered blind (1 yr), hearing difficulty requiring hearing aid, obesity (infancy), hyperlipidemia (18 yr), DM (18 yr).
ALSUK11	Exon 8 Exon 8	**c.4147_4150del** **c.4147_4150del**	p.(Ser1383Asnfs*19) p.(Ser1383Asnfs*19)	F	Impaired vision (infancy), obesity (infancy), hyperlipidemia, DM (8 yr), raised liver enzymes, microcephaly.
ALSUK12	Exon 8 Exon 8 Intron 9	**c.3392C>G** **c.3392C>G** **c.(7677+1_7678‐1)_(10387+1_10388‐1)del**	**p.(Ala1131Gly)** **p.(Ala1131Gly)** p.(Gly2560Serfs*46)	F	Impaired vision (severe), hearing difficulty requiring hearing aid, heart abnormality (infancy), obesity (infancy), hyperlipidemia, NAFLD, abnormal kidney function, kyphoscoliosis, chronic chest infections.
ALSUK13	Exon 8 Exon 8	**c.6532C>T** c.11107C>T	p.(Gln2178*) p.(Arg3703*)	M	Impaired vision (severe), hearing difficulty requiring hearing aid, heart defect (infancy), obesity (infancy), DM (18 yr), NALFD, raised creatinine (81 μmol/L).
ALSUK14	Exon 8 Exon 8	c.6829C>T c.9541C>T	p.(Arg2277*) p.(Arg3181*)	M	Impaired vision (severe), obesity, NAFLD
ALSUK15	Exon 8 Exon 8	c.6829C>T c.9541C>T	p.(Arg2277*) p.(Arg3181*)	F	Impaired vision (severe), hearing difficulty (infancy) requiring hearing aid, obesity (infancy), NAFLD, bladder dysfunction, gastro‐esophageal reflux, kyphoscoliosis.
ALSUK16	Exon 8 Exon 8	**c.6901del** c.11449C>T	p.(Val2301Trpfs*43) p.(Gln3817*)	M	Impaired vision (severe), hearing difficulty (infancy) requiring hearing aid, obesity (infancy), hyperlipidemia, NAFLD, abnormal kidney function, kyphoscoliosis (20 yr), hypogonadism.
SLO68‐13	Exon 8 Exon 16	c.4156dupA c.11207C>A	p.(Thr1386Asnfs*15) p.(Ser3736*)	F	Impaired vision, obesity, impaired glucose tolerance (13 yr), bilateral macular hypoplasia, bilateral cataract, microcrania, psychomotor delay.
SLO301‐11	Exon 10 Exon 10	**c.8456_8817del** **c.8456_8817del**	p.(Thr2819Argfs*29) p.(Thr2819Argfs*29)	F	Obesity, dilated cardiomyopathy, OA, retinal dystrophy, sensorineural hearing loss.

F, female; M, male; DM, diabetes mellitus; OA, optic atrophy; NAFLD, nonalcoholic fatty liver disease; yr, year.

*Notes*: Novel variants are in bold.

a Nucleotide numbering: +1 is A of ATG start codon (NCBI Reference Sequence NM_015120.4).

**Table 3:** Genetic and clinical finding in Wolfram syndrome patients

Patient/ethnicity	Location	Nucleotide change^a^	Protein change	Gender	Clinical findings
WSUK‐1	Exon 4	**c.334C>T**	p.(Gln112*)	M	DM 5 yr, OA 11 yr, hearing loss 8 yr, DI 26 yr, learning difficulties.
Middle Eastern	Exon 4	**c.334C>T**	p.(Gln112*)		
WSUK‐2.1	Exon 8	c.1549delC	p.(Arg517Alafs*5)	F	DM, OA, DI, hearing loss.
Caucasian	Exon 8	**c.2033G>A**	p.(Trp678*)		
WSUK‐2.2	Exon 8	c.1549delC	Arg517Alafs	F	DM, OA, DI, hearing loss, psychiatric disorder.
Caucasian	Exon 8	**c.2033G>A**	p.(Trp678*)		
WSUK‐3.1	Exon 8	c.2146G>A	p.(Ala716Thr)	M	DM 3 yr, OA 9 yr, hearing loss.
Caucasian	Exon 8	c.2648_2651del	p.(Phe883Serfs*68)		
WSUK‐3.2	Exon 8	c.2146GA	p.(Ala716Thr)	M	DM 5 yr, OA 23 yr, psychiatric problems.
Caucasian	Exon 8	c.2648_2651del	p.(Phe883Serfs*68)		
WSUK‐4.1	Exon 8	c.1525_1539del	p.(Val509_Tyr513del)	M	DM, OA 8 yr, DI 11 yr, bladder dysfunction, learning difficulties.
Middle Eastern		c.1525_1539del	p.(Val509_Tyr513del)		
WSUK‐4.2	Exon 8	c.1525_1539del	p.(Val509_Tyr513del)	F	DM, OA, DI 7 yr.
Middle Eastern	Exon 8	c.1525_1539del	p.(Val509_Tyr513del)		
WSUK‐5	Exon 8	**c.911_914dup**	p.(Met306*)	F	DM 5 yr, OA 6 yr, hearing loss (all frequencies) 4 yr, DI 16 yr, bladder dysfunction.
Caucasia	Exon 8	c.1994G>A	p.(Trp648*)		
WSUK‐6	Exon 8	c.2051C>T	p.(Ala684Val)	F	OA (mild), hearing loss 5 yr, no DM.
Caucasian	Exon 8	c.2452C>T	p.(Arg818Cys)		
WSUK‐7	Exon 5	c.505G>A	p.(Glu169Lys)	F	DM 4 yr, OA, DI 4 yr, tinnitus.
Caucasian	Exon 8	c.1558C>T	p.(Gln520*)		
WSUK‐8	Exon 4	**c.334C>T**	p.(Gln112*)	F	DM, OA, DI, hearing loss, sleep apnea, weak bones, learning difficulties.
Middle Eastern	Exon 4	**c.334C>T**	p.(Gln112*)		
WSUK‐9	Exon 4	c.409_424dup	p.(Val142Glyfs*110)	F	DM 7 yr, OA 6 yr, DI, hearing loss, bladder dysfunction, impaired renal function.
Caucasian	Exon 8	c.2262_2263del	p.(Cys755Serfs*3)		
WSUK‐10	Exon 8	c.1504_1527dup	p.(Ser503_Val509dup)	M	DM 13 yr, OA 15 yr, bladder dysfunction.
Caucasian	Exon 8	c.2262_2263del	p.(Cys755Serfs*3)		
WSUK‐11	Exon 8	**c.1338C>A**	**p.(Ser446Arg)**	F	DM 5.5 yr, OA 5.5 yr, hearing loss 3.5 yr.
Caucasian	Exon 8	c.2327A>T	p.(Glu776Val)		
WSUK‐12	Exon 8	**c.1283C>G**	**p.(Pro428Arg)**	F	DM 13 yr, OA 13 yr, hearing loss (high frequency), oral pharyngeal dysphasia, bladder dysfunction, psychiatric disorder.
Caucasian	Exon 8	**c.2319C>G**	p.(Tyr773*)		
WSUK‐13.1	Exon 8	c.1401_1403del	p.(Leu468del)	M	DM 4 yr, OA, DI, hearing loss, neuropathic bladder.
Middle Eastern	Exon 8	c.1401_1403del	p.(Leu468del)		
					
WSUK‐13.2	Exon 8	c.1401_1403del	p.(Leu468del)	M	DM 7 yr, OA 7 yr, neuropathic bladder, psychiatric disorder.
Middle Eastern	Exon 8	c.1401_1403del	p.(Leu468del)		
WSUK‐13.3	Exon 8	c.1401_1403del	p.(Leu468del)	F	DM 5 yr, OA, hearing loss (all freq.), ataxia, psychiatric disorder.
Middle Eastern	Exon 8	c.1401_1403del	p.(Leu468del)		
WSUK‐14	Exon 8	c.906C>A	p.(Tyr302*)	F	DM, OA, bulbar palsy with recurrent choking episodes, sleep apnea, bladder dysfunction, cerebellar pontine hypoplasia.
Caucasian	Exon 8	c.2648_2651del	p.(Phe883Serfs*68)		
WSUK‐15	Exon 8	c.2099G>A	p.(Trp700*)	M	DM 6 yr, OA 9 yr, DI, hearing loss (high frequency), bladder dysfunction.
South European	Exon 8	c.2099G>A	p.(Trp700*)		
	Exon 8				
WSUK‐16	Exon 5	c.505G>A	p.(Glu169Lys)	M	DM 14 yr, OA, DI, hearing loss (high frequency), neurogenic bladder.
Caucasian	Exon 8	**c.874C>A**	**p.(Pro292Thr)**		
WSUK‐17	Exon 4	**c.334C>T**	p.(Gln112*)	M	DM 2 yr, OA 20 yr, DI, hearing loss 6 yr, neurogenic bladder, learning difficulties, psychiatric disorder.
Middle Eastern	Exon 4	**c.334C>T**	p.(Gln112*)		
WSUK‐18	Exon 8	c.2002C>T	p.(Gln668*)	F	DM 3 yr, OA 11 yr, hearing loss (high frequency, mild).
Caucasian	Exon 8	**c.2080G>T**	p.(Glu694*)		
WSUK‐19	Exon 8	**c.1727_1744del**	**p.(Gly576_Gly581del)**	F	DM 8 yr, OA 34 yr, hearing loss 26 yr, bladder dysfunction.
Middle Eastern	Exon 8	**c.1727_1744del**	**p.(Gly576_Gly581del)**		
WSUK‐20.1	Exon 8	c.2654C>T	p.(Pro885Leu)	M	DM 4 yr, OA 9 yr, DI 24 yr, deteriorating balance and mobility, choking episodes, psychiatric disorder.
Middle Eastern	Exon 8	c.2654C>T	p.(Pro885Leu)		
WSUK‐20.2	Exon 8	c.2654C>T	p.(Pro885Leu)	M	DM 5 yr, OA 11 yr, DI 20 yr, psychiatric disorder.
Middle Eastern	Exon 8	c.2654C>T	p.(Pro885Leu)		
WSUK‐21	Exon 8	**c.1434del**	p.(Trp478Cysfs*4)	F	DM 6 yr, OA, DI, bladder dysfunction, psychiatric disorder (mild).
Caucasian	Exon 8	**c.2425G>T**	p.(Glu809*)		
WSUK‐22	Exon 8	c.1549C>T	p.(Arg517Cys)	F	DM 3yr, OA 5 yr, DI 8 yr, hearing loss, ataxia, chronic fatigue syndrome, psychiatric disorders.
Caucasian	Exon 8	**c.1682T>G**	**p.(Ile561Ser)**		
	Exon 8	**c.1775T>C**	**p.(Leu592Pro)**		
	Exon 8	**c.1944G>A**	p.(Trp648*)		
WSUK‐23.1^b^	Exon 5	c.505G>A	p.(Glu169Lys)	M	DM 11 yr, OA 12 yr, DI, hearing loss 13 yr, bladder dysfunction, cerebellar signs.
Caucasian	Exon 7	c.817G>T	p.(Glu273*)		
WSUK‐23.2^b^	Exon 5	c.505G>A	p.(Glu169Lys)	F	DM 11 yr, OA 12 yr, DI, hearing loss 13 yr.
Caucasian	Exon 7	c.817G>T	p.(Glu273*)		
WSUK‐24	Exon 8	c.1433G>A	p.(Trp478*)	F	DM 10 yr, OA 11 yr, DI, bladder dysfunction, delayed puberty, pharyngeal dysphasia, cerebellar dysfunction.
Caucasian	Exon 8	c.2648_2651del	p.(Phe883Serfs*68)		
WSUK‐25	Exon 8	c.1049_1051del	p.(Phe350del)	M	DM 13 yr, DI 16 yr, bladder dysfunction.
Caucasian	Exon 8	c.2206G>A	p.(Gly736Ser)		
WSUK‐26	Exon 8	c.1309G>C	p.(Gly437Arg)	F	DM, OA, DI
Caucasian	Exon 8	**c.1434del**	p.(Trp478Cysfs*4)		
WSUK‐27	Exon 8	c.1230_1233del	p.(Val412Serfs*29)	F	DM 5 yr, poor night vision, DI, hearing loss 15 yr, bilateral cataract, microalbuminuria, cerebellar signs (mild).
Caucasian	Exon 8	c.1243_1245del	p.(Val415del)		
WSUK‐28.1	Exon 8	**c.2006A>C**	**p.(Tyr669Ser)**	F	DM, OA, DI, hearing loss 7 yr, congenital hypothyroidism, diabetic retinopathy, neuropathic bladder, psychiatric disorder.
Caucasian	Exon 8	**c.2006A>C**	**p.(Tyr669Ser)**		
WSUK‐28.2	Exon 8	**c.2006A>C**	**p.(Tyr669Ser)**	M	DM 4 yr, OA, DI, diabetic retinopathy, mild ataxia, microcephaly.
Caucasian	Exon 8	**c.2006A>C**	**p.(Tyr669Ser)**		
WSUK‐29	Exon 8	c.2099G>A	p.(Trp700*)	M	DM, OA, hearing loss (high frequency).
South European	Exon 8	c.2099G>A	p.(Trp700*)		
WSUK‐30	Exon 8	c.1096C>T	p.(Gln366*)	F	DM, OA
South European	Exon 8	c.1672C>T	p.(Arg558Cys)		
WSUK‐31.1	Exon 4	**c.334C>T**	p.(Gln112*)	F	p.(Gln112*)
Middle Eastern	Exon 4	**c.334C>T**	p.(Gln112*)		
WSUK‐31.2	Exon 4	**c.334C>T**	p.(Gln112*)	M	DM 7 yr, OA 9 yr, coeliac disease, resting hand tremors.
Middle Eastern	Exon 4	**c.334C>T**	p.(Gln112*)		
WSUK‐32.1	Exon 8	c.2643_2646del	p.(Phe882Serfs*69)	F	DM 4.5 yr, OA 9 yr, DI 13 yr.
Middle Eastern	Exon 8	c.2643_2646del	p.(Phe882Serfs*69)		
WSUK‐32.2	Exon 8	c.2643_2646del	p.(Phe882Serfs*69)	M	DM 2.5 yr, bladder dysfunction.
Middle Eastern	Exon 8	c.2643_2646del	p.(Phe882Serfs*69)		
WSUK‐33	Exon 8	**c.958_961delinsTCC**	p.(Pro320Serfs*39)	M	DM, OA, hearing loss, bladder dysfunction, primary testicular atrophy, psychiatric disorder.
Middle Eastern	Exon 8	**c.958_961delinsTCC**	p.(Pro320Serfs*39)		
WSUK‐34	Exon 4	**c.334C>T**	p.(Gln112*)	M	DM, OA, DI
Middle Eastern	Exon 4	**c.334C>T**	p.(Gln112*)		
WSUK‐35	Exon 8	c.2099G>A	p.(Trp700*)	F	DM 4 yr, OA 7 yr
South European	Exon 8	c.2099G>A	p.(Trp700*)		
WSUK‐36.1	Exon 8	**c.1549del**	p.(Arg517Alafs*5)	F	OA, hearing loss, neurogenic bladder, autonomic dysfunction.
Caucasian	Exon 8	c.1597C>T	p.(Pro533Ser)		
WSUK‐36.2	Exon 8	**c.1549del**	p.(Arg517Alafs*5)	M	OA, hearing loss (high frequency), urinary urgency, erectile dysfunction, restless leg syndrome.
Caucasian	Exon 8	c.1597C>T	p.(Pro533Ser)		
WSUK‐37	Exon 8	**c.1309G>C**	**p.(Gly437Arg)**	F	DM 8 yr, OA 22 yr, hearing loss, bladder dysfunction, ataxia, psychiatric disorder.
Caucasian	Exon 8	c.1699_1704del	p.(Leu567_Phe568del)		
WSUK‐38	Exon 5	c.605A>G	p.(Glu202Gly)	F	DM 14 yr, OA 14 yr, neurogenic bladder.
Caucasian	Exon 7	c.817G>T	p.(Glu273*)		
WSUK‐39	Exon 7	c.817G>T	p.(Glu273*)	M	DM 7 yr, OA 7 yr, DI 8 yr, hearing loss (high frequency) 6 yr, bladder dysfunction.
Caucasian	Exon 8	c.1504_1527dup	p.(Ser502_Val509dup)		
WSUK‐40	Exon 4	c.376G>A	p.(Ala126Thr)	M	DM 10 yr, OA 8 yr, hearing loss (high freq.) 11 yr, psychiatric disorder.
Caucasian	Exon 8	c.1885C>T	p.(Arg629Trp)		
WSUK‐41	Exon 8	**c.1529_1543del**	**p.(Tyr510_Leu514del)**	M	DM 2 yr, OA 7 yr, DI, mild cerebellar dysfunction.
Caucasian	Exon 8	c.2254G>T	p.(Glu752*)		
WSUK‐42	Exon 8	c.2648_2651del	p.(Phe883Serfs*68)	F	DM 10 yr.
Caucasian	Exon 8	c.2648_2651del	p.(Phe883Serfs*68)		
WSUK‐43	Exon 8	c.2648_2651del	p.(Phe883Serfs*68)	F	DM 11 yr, OA 10 yr, learning difficulties, reduced white matter on MRI brain scan.
Caucasian	Intron 1	**c. ‐6G>T**	**p.?**		
WSUK‐44	Exon 8	c.937C>T	p.(His313Tyr)	M	DM 1.5 yr, OA (mild), congenital hearing loss.
Middle Eastern	Intron 1	**c.‐184_‐179dupTGCCCC**	**p.?**		
WSUK‐45	Exon 8	**c.1153G>A** c	**p.(Glu385Lys)**	F	DM 9 yr, OA 4 yr, congenital hearing loss.
Caucasian					
WSUK‐46	Exon 8	c.2590G>A^c^	p.(Glu864Lys)	M	
Caucasian					OA 10 yr, congenital hearing loss.
WSUK‐47	Exon 8	c.937C>T^c^	p.(His313Tyr)	F	OA, DM, hearing loss, short stature
Caucasian					
WSUK‐48	Exon 8	**c.977C>T**	**p.(Ala326Val)**	F	OA, DM
Caucasian	Exon 8	c.1309G>C	p.(Gly437Arg)		

F, female; M, male; DM, diabetes mellitus; OA, optic atrophy; DI, diabetes insipidus; yr, year.

*Notes*: Novel variants are in bold.

a Nucleotide numbering: +1 is A of ATG start codon (NCBI Reference Sequence NM_006005.3).

b Twin.

c Only heterozygous variant identified.

*ALMS1*: six nonsense variants, c.800G>A (p.Trp267*), c.1011_1012del (p.Cys337*) in exon 5, c.4321C>T (p.Gln1441*), c.6325G>T (p.Glu2109*), c.6532C>T (p.Gln2178*) in exon 8, and c.9258dup (p.Asp3087*) in exon 10, have been identified. This report expands the spectrum of *ALMS1* variants in exon 5 first described by Marshall et al. (2015). Nine novel variants causing frameshifts are predicted to result in premature stop codon, and protein truncation have been identified in exon 8, intron 9, exon 10, and exon 18. Six frameshift variants identified in exon 8 are: c.224dup (p.Thr742Asnfs*2), c.4025_4026delinsA (p.Gly1342Glufs*18), c.4053_4054del (p.His1351Glnfs*5), c.4147_4150del (p.Ser1383Asnfs*19), c.5081del (p.Pro1692Leufs*39), and c.6901del (p.Val2301Trpfs*43). The c.(7677+1_7678‐1)_(10387+1_10388‐1)del (p.Gly2560Serfs*46) identified in intron 9, a 362‐bp deletion c.845brk6_8817del (p.Thr2819Argfs*29) was identified in exon 10 and c.11738dup (p.Ser3914Lysfs*6) was found in exon 18.

The significance of the missense variant c.3392C>G (p.Ala1131Gly) was unclear, although it is predicted to be probably damaging by the PolyPhen‐2 algorithm. In the patient, this variant occurs in the homozygous state together with a homozygous pathogenic frameshift‐causing deletion (c.(7677+1_7678‐1)_(10387+1_10388‐1)del; p.Gly2560Serfs*46). Homozygous missense variant c.4225G>A (p.Val1409Ile; rs200529564) identified in exon 8 was likely benign, as the patient also carries the homozygous novel pathogenic variant c.5081del (p.Pro1692Leufs*39).

*WFS1*: twenty‐three novel variants in the *WFS1* gene have been identified: two in intron 1, one in exon 4, and the rest are in exon 8 (Table 3). Seven of these are nonsense variants causing early termination of the Wolframin protein, two are causing frameshift, three result in in‐frame deletions, nine are missense/nonsynonymous variants, and two affect splice/regulatory regions.

A novel homozygous nonsense variant c.334C>T; p.Gln112* was identified in six individuals from five different families of Middle Eastern origin. Other novel nonsense variants identified are c.911_914dup; p.Met306*, c.977C>T; p.Ala326Val, c.1944G>A; p.Trp648*, c.2033G>A; p.Trp678*, c.2080G>T; p.Glu694*, c.2319C>G; p.Tyr773*, and c.2425G>T; p.Glu809*. In addition to two novel frameshift variants, c.1434del; p.Trp478Cysfs*4 and c.958_962delinsTCC; p.Pro320Serfs*39, we also identified three novel in‐frame deletions in our cohort: c.1529_1543del; p.Tyr510_Leu514del, c.1699_1704del; p.Leu567_Phe568del, and c.1727_1744del; p.Gly576_Gly581del.

Novel missense variants p.Pro292Thr (c.874C>T; rs746923441), p.Pro428Arg (c.1283C>G), p.Ser446Arg (c.1338C>A), p.Pro533Ser (c.1597C>T; rs146132083), and p.Tyr669Ser (c.2006A>C) are predicted to be damaging/probably damaging by both SIFT and PolyPhen‐2, segregated with disease phenotypes, and are classified as pathogenic. Four of the novel missense variants, p.Ala326Val (c.977C>T), p.Glu385Lys (c.1153G>A; rs71524353), and p.Leu592Pro (c.1775T>C) are predicted to be damaging/probably damaging by one of the prediction algorithms and are classified as likely pathogenic. The significance of p.Ile561Ser (c.1682T>G; rs776993839), predicted to be damaging by SIFT, is yet uncertain. In patient WSUK‐45, who presented with congenital hearing loss, childhood OA, and juvenile DM, the p.Glu385Lys was present in a heterozygous state with no other *WFS1* mutation. This patient also presented with an *OPA1* pathogenic mutation (duplication of exons 4–8) inherited from her mother and maternal grandfather. Both have OA with *OPA1* and *WFS1* variants. Bonnycastle et al. (2013) identified a single‐missense *WFS1* mutation (p.Trp314Arg) segregating with diabetes in a multigeneration Finnish family. It is possible that the c.1153G>A; p.Glu385Lys also has a dominant effect and is responsible for the diabetes phenotype in our WSUK‐45 patient. Although the patient's mother also presented with insulin‐dependent diabetes, no information is available on the diabetes status of the maternal grandfather to further support this claim. In our cohort, two families are also identified with a single nonsynonymous pathogenic *WFS1* variant, interestingly both involving substitution of amino acid glutamate to lysine (p.Glu385Lys and p.Glu864Lys).

The effect of the novel intron 1 variants, the c.‐6G>T substitution, and the c. ‐184_‐179dup duplication is not yet clear. Fibroblasts from the patient harboring this variant and a pathogenic c.937C>T; p.His313Tyr shows significant reduction of *WFS1* expression in Western blot analysis (data not shown). However, a single c.937C>T; p.His313Tyr mutation has been shown to be capable of causing WS (Hansen et al., 2005) and inducing ER stress (Bonnycastle et al., 2013). One of our patients (WSUK‐47) was also identified with the p.His313Tyr variant without the presence of another pathogenic *WFS1* variant.

### 3.3 *WFS1* genotype–phenotype analysis

We collected information on the age of onset of DM, OA, deafness, DI, and other reported clinical features from patients in the database and categorized the disease by phenotype and genotype.

Disease phenotype was classified as: (1) WS, defined by biallelic inheritance of *WFS1* variants and the presence of two major features (DM and OA) at any age of onset with/without other associated clinical features (deafness, DI, neurological disorders), or the presence of one major feature accompanied by at least two associated clinical features; (2) *WFS1*‐related disorders (recessive form), defined by biallelic inheritance of *WFS1* variants and the presence of one major feature (DM or OA) with none or only one associated feature; and (3) *WFS1*‐related disorders (dominant form), defined by dominant inheritance of a *WFS1* variant and the presence of one or more clinical features (sensorineural deafness, DM, OA, DI, cataract).

**Figure 1:**
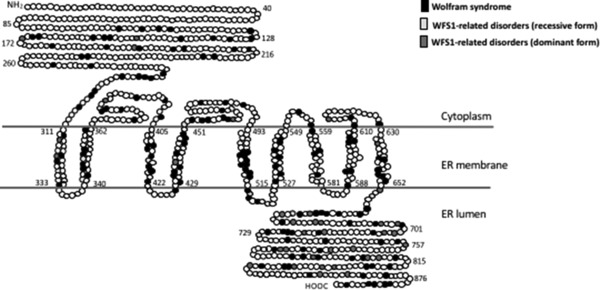
WFS1 variant distribution based on disease phenotype. Position of the amino acid involved in the disease phenotype is indicated by different shades. Position of the transmembrane regions were predicted based on TMHMM (Krogh, Larsson, von Heijne, & Sonnhammer, 2001) and SMART (Letunic, Doerks, & Bork, 2015). ER, endoplasmic reticulum

The patients’ genotypes were classified into two variant groups: group 1 are variants predicted to cause complete or partial loss of function (N‐terminal nonsense and frameshifts, splice‐site variants predicted to cause exon skipping/deletions; C‐terminal nonsense and frameshift; N‐terminal small in‐frame deletions/duplications/insertions/indels); or compound heterozygous where one variant is predicted to cause complete and the other a partial loss of function. Group 2 are variants predicted to cause minor loss of function (missense, C‐terminal small in‐frame deletions/duplications/insertions/indels) or compound heterozygous for a variant predicted to cause partial and minor loss of function (Supp. Table S1). We defined the *WFS1* N‐terminal as amino acids at positions 1–652 (cytosolic and transmembrane domain) and the C‐terminal as amino acids at positions 653–890 (ER lumen) (Fig. 1).

Patients whose phenotype could clearly be identified were then assigned into respective genotype and phenotype categories (Supp. Table S1). Vassar Stats Clinical Calculator 1 (vassarstats.net) was used to estimate population prevalence, sensitivity, specificity, predictive values, and likelihood ratios. The age of onset of DM, OA, and DI between genotypic groups were compared using ANOVA. From 448 patients analyzed, 301 belonged to group 1 and 147 to group 2 genotypes. In patients with group 1 genotype, 295 have the WS phenotype and six have a recessive form of *WFS1*‐related disorder. In patients with group 2 genotype, 78 have WS phenotype, eight have recessive forms of *WFS1*‐related disorders, and 61 patients presented with dominant forms of *WFS1*‐related disorders (Table 4). The classification of a group 1 genotype is highly sensitive (75%–83%) and specific (83%–97%) in predicting a WS phenotype with a positive predictive value of 95%–99%. The classification of a group 2 genotype has a modest sensitivity (30%–81%) and specificity (63%–72%) in predicting recessive *WFS1*‐related disorders; however, it has high sensitivity (93%–100%) and specificity (73%–82%) in predicting the dominant form of *WFS1*‐related disorders (Table 5). Six of the patients harboring group 1 genotypes presented without a classic WS phenotypes (no reported OA at adulthood or during data collection). Four of these patients are brothers from a Latin American family and two are from different UK‐White European families. In these cases, we speculate that genetic and environmental interactions may contribute to variable expressivity.

**Table 4:** Number of patient classified according to genotype and phenotype

Phenotype	Group 1	Group 2	Total
Wolfram syndrome	295	78	373
WFS1‐related disorders (recessive form)	6	8	14
WFS1‐related disorders (dominant form)	0	61	61
Total	301	147	448

*Notes*: Group 1: variants predicted to cause complete or partial loss of function (N‐terminal nonsense and frameshifts, splice‐site variants predicted to cause exon skipping/deletions; C‐terminal nonsense and frameshift; N‐terminal small in‐frame deletions/duplications/insertions/indels); or compound heterozygous where one variant is predicted to cause complete and the other a partial loss of function.

Group 2: variants predicted to cause minor loss of function (missense, C‐terminal small in‐frame deletions/duplications/insertions/indels) or compound heterozygous for a variant predicted to cause partial and minor loss of function. See Supp. Table S1 for detail.

**Table 5:** Sensitivity and specificity of WFS1 genotype to predict phenotype

Phenotype	Genotype	Sensitivity (95% CI)	Specificity (95% CI)	Positive predictive value (95% CI)	Negative predictive value (95% CI)
Wolfram syndrome	Group 1	79 (75, 83)	92 (83, 97)	98 (95, 99)	47 (39, 55)
	Group 2	21 (17, 25)	8 (3, 17)	53 (45, 61)	2 (1, 5)
WFS1‐related disorders (recessive form)	Group 1	43 (19, 70)	32 (28, 37)	2 (1, 5)	95 (89, 97)
	Group 2	57 (30,81)	68 (63, 72)	5 (3, 11)	98 (95, 99)
WFS1‐related disorders (dominant form)	Group 1	0 (0, 7)	22 (18, 27)	0 (0, 2)	58 (50, 66)
	Group 2	100 (93, 100)	78 (73, 82)	42 (34, 50)	100 (98, 100)

CI, confidence interval.

*Notes*: Group 1: variants predicted to cause complete or partial loss of function (N‐terminal nonsense and frameshifts, splice‐site variants predicted to cause exon skipping/deletions; C‐terminal nonsense and frameshift; N‐terminal small in‐frame deletions/duplications/insertions/indels); or compound heterozygous where one variant is predicted to cause complete and the other a partial loss of function.

Group 2: variants predicted to cause minor loss of function (missense, C‐terminal small in‐frame deletions/duplications/insertions/indels) or compound heterozygous for a variant predicted to cause partial and minor loss of function.

Sensitivity, specificity, positive predictive value, and negative predictive value was calculated using VassarStats Clinical calculator 1 (www.vassarstats.net).

Comparison of the age of onset of DM and OA in group 1 and group 2 genotypes revealed a highly significant difference in phenotypes between the two groups. The mean age of onset of DM was 6.3 ± 3.5 years in patients with group 1 genotypes and 12.0 ± 9.9 years in individuals with group 2 genotypes (*P* < 0.0001), whereas the mean age of onset of OA was 11.7 ± 5.7 years in individuals with group 1 genotypes and 15.8 ±11.4 years in individuals carrying group 2 genotypes (*P* = 0.0023). A significant difference in the age of onset of DI was also observed between individuals carrying group 1 and group 2 genotypes. The mean age of onset of DI was 13.9 ± 6 years and 18.0 ± 10 years in group 1 and group 2 genotypes (*P* = 0.047), respectively (Table 6). Rohayem et al. (2011) and de Heredia, Clèries, and Nunes (2013) also showed significant differences in the age of onset of DM and DI among patients carrying predicted complete, partial, or minor loss‐of‐function mutation. However, due to differences in genotypic classification used by these authors, the mean age of onset of DM and DI cannot be directly compared. It has been previously reported that some patients harboring a homozygous frameshift variant in the C‐terminal end of *WFS1* tend to have a delayed onset of OA (Zalloua et al., 2008). We therefore analyzed 19 patients carrying homozygous frameshift variants in the C‐terminal of *WFS1* (patients 265, 271–280, 285, 290, 295–298, 300, and 301 in Supp. Table S1), and 33 patients harboring a homozygous frameshift variant in the N‐terminal region (patients 14–21, 58, 63, 87–91, 104, 112, 116, 118, 119, 123, 135, 198, 202, 235, 243–247, and 252–254 in Supp. Table S1). There is a slight difference in the age of onset of OA in patients with homozygous frameshift C‐terminal variant compared with the age of OA onset in patients with homozygous frameshift N‐terminal variants (13.2 ± 5 years and 11.2 ± 6.1 years, respectively). However, this is not statistically significant. Variants associated with a WS phenotype were distributed in both outside and inside the transmembrane region, whereas variants involved in the dominant form of *WFS1*‐related disorder were mainly located at the C‐terminal end of the protein (Fig. 1).

**Table 6:** Age of onset of diabetes mellitus, optic atrophy, hearing loss, and diabetes insipidus based on genotype classification

Genotype	Diabetes mellitus (Mean ± SD)	Optic atrophy (Mean ± SD)	Deafness (Mean ± SD)	Diabetes insipidus (Mean ± SD)
Group 1	6.3 ± 3.5 years	11.7 ± 5.7 years	14.4 ± 7.2 years	13.9 ± 6.7 years
	*n* = 300	*n* = 249	*n* = 142	*n* = 114
Group 2	12.0 ± 9.9 years	15.8 ± 11.4 years	18.0 ± 13.7 years	18.0 ± 10.2 years
	*n* = 90	*n* = 81	*n* = 37	*n* = 29
*P* (*t*‐test)	<0.0001	0.0021	0.125	0.047

*Notes*: Group 1: variants predicted to cause complete or partial loss of function (N‐terminal nonsense and frameshifts, splice‐site variants predicted to cause exon skipping/deletions; C‐terminal nonsense and frameshift; N‐terminal small in‐frame deletions/duplications/insertions/indels); or compound heterozygous where one variant is predicted to cause complete and the other a partial loss of function.

Group 2: variants predicted to cause minor loss of function (missense, C‐terminal small in‐frame deletions/duplications/insertions/indels) or compound heterozygous for a variant predicted to cause partial and minor loss of function.

### 3.4 Future prospects and database update

The clinical overlaps and complexity exhibited by the syndromes mentioned in this report may lead to delayed or misdiagnosis. We have demonstrated that a more detailed description of clinical phenotypes in the patients coupled with genotype information can provide insight into genotype–phenotype correlations of these syndromes. Unfortunately, the clinical phenotypes are difficult to access, not always available, and can be unreliable sometimes in terms of age of onset. We hope that the information available for some of the patients in our databases will allow for better understanding of the disease and reliable genetic counselling for the patients and their families. Ultimately, functional studies of the variants will be necessary to further our understanding of disease mechanisms that will lead to the development of personalized therapies.

The EURO‐WABB LOVD locus‐specific databases for *ALMS1/WFS1/CISD2/ SLC19A2* have been available online since 2012 and have received submissions of variants identified in patients. Future contributors can submit their variants online or by contacting and providing curators with the necessary information. When referring to the EURO‐WABB *ALMS1/WFS1/CISD2/ SLC19A2* LOVD databases, we kindly ask users to cite this article.

## Supporting information

Supp. Table S1 Patients included in the genotype‐phenotype analysis.
